# How does literacy break mirror invariance in the visual system?

**DOI:** 10.3389/fpsyg.2014.00703

**Published:** 2014-07-10

**Authors:** Felipe Pegado, Kimihiro Nakamura, Thomas Hannagan

**Affiliations:** ^1^Laboratory of Biological Psychology, Department of Psychology and Educational Sciences, KU Leuven UniversityLeuven, Belgium; ^2^Human Brain Research Center, Graduate School of Medicine, Kyoto UniversityKyoto, Japan; ^3^Laboratoire de Psychologie Cognitive, Fédération de Recherche 3C, Centre National de la Recherche Scientifique, Aix-Marseille UniversityMarseille, France

**Keywords:** multisensory, multi-system, reading, writing, literacy, alphabetization, mirror invariance, mirror discrimination

A growing literature has been showing a profound impact of alphabetization at several levels of the visual system, including the primary visual cortex (Szwed et al., 2014) and higher-order ventral and dorsal visual areas (Carreiras et al., 2009; Dehaene et al., 2010). Importantly, in typical alphabetization courses, learning to read is not isolated but instead combined with both learning to write and learning to segment the spoken language, relating all these different representations to each other. Indeed, learning to write and to pronounce the elementary sounds of language promotes additional mapping between the visual and motor systems by linking visual representations of letters and motor plans for handwriting and speech production. Thus, besides the already recognized influence of the phonological system, the potential influence from other neural systems in the functioning of the visual system seems to be relatively neglected. In this opinion paper we highlight the importance of multi-systems interplay during literacy acquisition, focusing on the question of how literacy breaks mirror invariance in the visual system. Specifically, we argue for a large contribution of top-down inputs from phonological, handwriting and articulatory representations toward the ventral visual cortex during the development of the visual word form system, which then plays a pivotal role in mirror discrimination of letters in literate individuals.

## How phonology affects visual representations for reading

A key aspect of alphabetization is to set in place the audio-visual mapping known as “phoneme-grapheme correspondence,” whereby elementary sounds of language (i.e., phonemes) are linked to visual representations of them (i.e., graphemes) (Frith, 1986). This correspondence is progressively acquired and becomes automatized typically after 3–4 years of training (Nicolson et al., 2001; Van Atteveldt et al., 2004; Lachmann and van Leeuwen, 2008; Dehaene et al., 2010; Lachmann et al., in this special issue). Illiterates, who do not learn this audio-visual correspondence, are unable to show “phonological awareness” (i.e., the ability to consciously manipulate language sounds) at the phonemic level (Morais et al., 1979; Morais and Kolinsky, 1994), presenting different visual analytical characteristics (Lachmann et al., 2012; Fernandes et al., 2014). Accordingly, activations in phonological areas increases in proportion to the literacy level of participants, e.g., *planum temporale* responses to auditory sentences and left superior temporal sulcus responses to visual presentations of written sentences (Dehaene et al., 2010). These results therefore suggest an important link between the visual and auditory systems created by literacy training. Indeed, the reciprocal inter-regional coupling between visual and auditory cortical areas may constitute a crucial component for fluent reading, since dyslexic children, who present slow reading, show reduced activations to speech sounds in the perisylvian language areas and ventral visual cortex including the Visual Word Form Area (VWFA) (Monzalvo et al., 2012).

## How writing affects visual representations for reading

In parallel, children (and adults) under alphabetization also learn to draw letters of the alphabet. Indeed, writing requires fine motor coordination of hand gestures, a process guided by online feedback from somatosensory and visual systems (Margolin, 1984). In particular, gestures of handwriting are thought to be represented in the dorsal part of the premotor cortex, rostral to the primary motor cortex responsible for hand movements, i.e., a region first coarsely described by Exner as the “graphic motor image center” (see Roux et al., 2010 for a review). Exner's area is known to be activated when participants write letters but not when they copy pseudoletters (Longcamp et al., 2003). Moreover, direct brain stimulation of the same region produces a specific inability to write (Roux et al., 2009). Importantly, this region is activated simply by visual presentations of handwritten stimuli (Longcamp et al., 2003, 2008), even when they are presented unconsciously (Nakamura et al., 2012). Additionally these activations take place in the premotor cortex contra-lateral to the dominant hand for writing (Longcamp et al., 2005). These results suggest that literacy training establishes a tight functional link between the visual and motor systems for reading and writing. In fact, it has been proposed that reading and writing rely on distributed and overlapping brain regions, each showing slightly different levels of activation depending on the nature of orthography (Nakamura et al., 2012). As for the reciprocal link between the visual and motor components of this reading network, brain-damaged patients and fMRI data from normal subjects consistently suggest that top-down activation of the posterior inferior temporal region constitutes a key component for both handwriting (Nakamura et al., 2002; Rapcsak and Beeson, 2004) and reading (Bitan et al., 2005; Nakamura et al., 2007).

## How speech production affects visual representations for reading

While the impact of auditory phonological inputs for literacy acquisition has been well demonstrated (e.g., phonological awareness studies), relatively less explored has been the connection between the speech production system and other systems during alphabetization. Indeed, although all alphabetizing children already speak fluently, an unusual segmentation and refinement of motor plans for speech production should be learned to pronounce isolated phonemes, allowing a multisensory association (explicitly or implicitly) of these new fine-grained phonatory representations with visual and auditory representations. One study has shown activation in a cortical region involved in speech production (Broca's area) in relation to handwriting learning and letter identification (Longcamp et al., 2008). In fluent readers, the inferior frontal area involved in speech production in one hand and the VWFA in another hand show fast and strong inter-regional coupling (Bitan et al., 2005), which operates even for unconsciously perceived words (Nakamura et al., 2007). This distant visual and articulatory link mediating print-to-sound mapping is probably established during the earliest phase of reading acquisition and serves as a crucial foundation for the development of a dedicated reading network (Brem et al., 2010).

## Literacy acquisition as a multi-system learning process: the example of mirror discrimination learning

Taken together, these studies converge to the idea that far fromiinfar from a unimodal training on visual recognition, literacy acquisition is an irreducibly multi-system learning process. This lead us to predict that as one becomes literate, the expertise acquired through a given modality is not restricted to it, but can have an impact on other neural systems.

Perhaps the most spectacular case in point, and the one we choose to focus on in this article, is the spontaneous link between the motor and visual systems during literacy acquisition. This link is revealed in the beginning of the alphabetization process by the classic emergence of spontaneous mirror writing, i.e., writing letters in both orientations indistinctly (Cornell, 1985). Indeed our primate visual system presents a mirror invariant representation of visual stimuli, which enables us to immediately recognize one image independently of left or right viewpoints (Rollenhagen and Olson, 2000; Vuilleumier et al., 2005; Biederman and Cooper, 2009). This generates a special difficulty to distinguish the left-right orientation of letters (e.g., b vs. d) (Orton, 1937; Corballis and Beale, 1976; Lachmann, 2002; Lachmann et al. in this special issue). One account for the emergence of mirror writing is that writing gestures can be “incorrectly” guided by mirror invariant visual representations of letters, a framework referred to as “perceptual confusion” (see Schott, 2007 for a review on this topic).

In complement, recent studies demonstrate that *after* literacy acquisition, mirror invariance is lost for letter strings (Kolinsky et al., 2011; Pegado et al., 2011, 2014) and that the VWFA shows mirror discrimination for letters (Pegado et al., 2011); see figure upper part. Interestingly, in this special issue, Nakamura and colleagues provide evidence for the causal role of the left occipito-temporal cortex (encompassing the VWFA) in mirror discrimination by using transcranial magnetic stimulation. However, it is still an open question whether this region becomes completely independent to discriminate the correct orientation of letters or if it still depends on inputs from phonological, gestural, and/or vocal representations.

## A multi-system model of mirror discrimination learning

**Figure 1:**
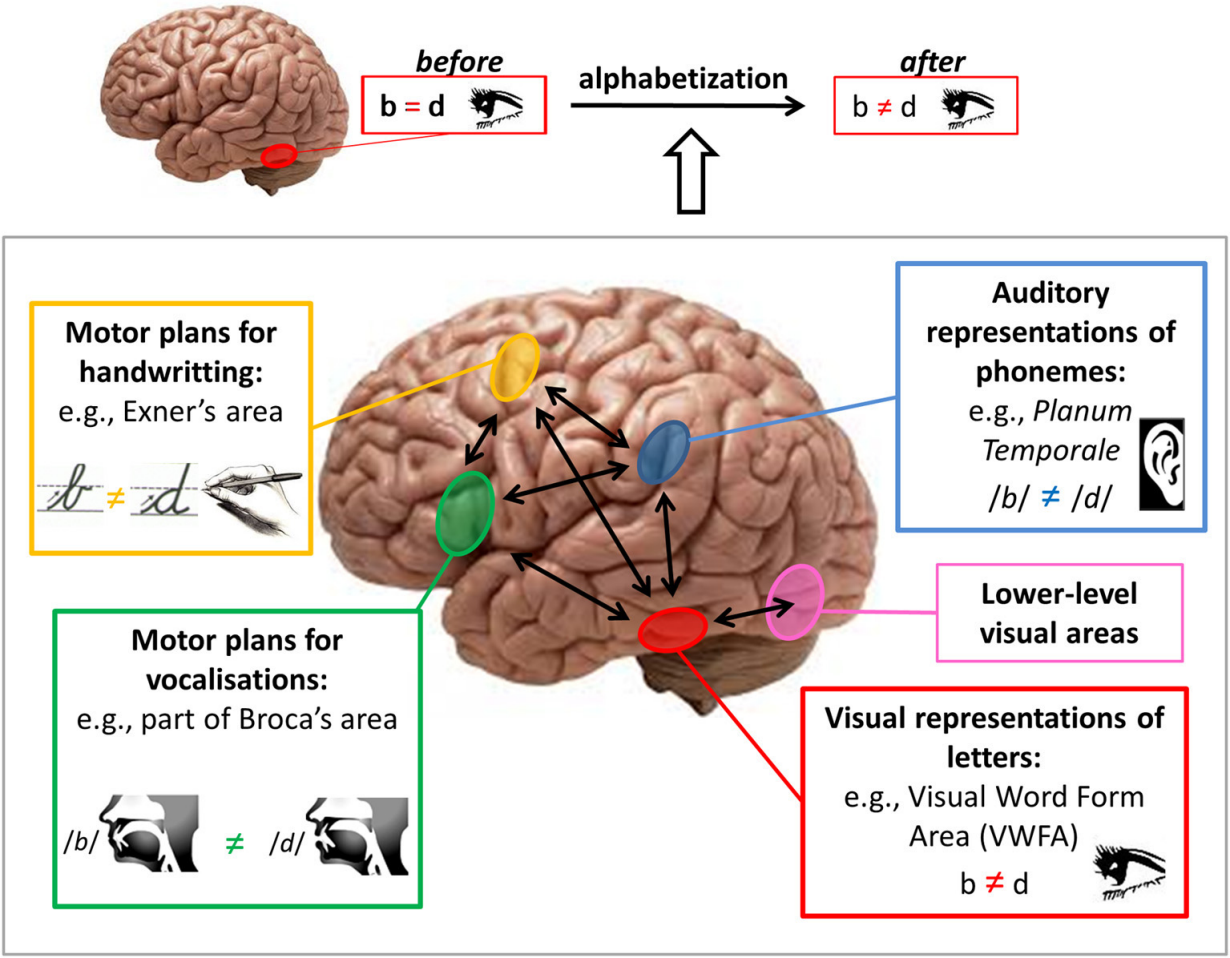
**Brain pathways for mirror discrimination learning during literacy acquisition**. Upper: The Visual Word Form Area [VWFA] (in red) presents mirror invariance before alphabetization and mirror discrimination for letters after alphabetization. Lower: During alphabetization, the VWFA can receive top-down inputs with discriminative information from phonological, gestural (handwriting) and speech production areas and bottom-up inputs from lower level visual areas. All these inputs can help the VWFA to discriminate between mirror representations, thus correctly identifying letters to enable a fluent reading.

How is mirror discrimination acquired *during* the process of literacy acquisition? Here we sketch a model that takes into account not only the multisensory nature of alphabetization but also the multi-systems interplay, i.e., how representations in one system could influence the functioning of another system (e.g., mirror invariance in the visual system). In Figure 1, we present the hypothetical “multi-system input model” for mirror-letters discrimination learning during literacy acquisition. In order to correctly and rapidly identify letters for a fluent reading, the VWFA (in red) should visually distinguish between mirror representations of letters (see figure upper part). Top-down inputs from phonological, handwriting and speech production representations can provide discriminative information to the VWFA, helping this area that presents intrinsic mirror invariance, to accomplish its task of letter identification. This process probably requires focused attention (not represented in the figure) during the learning process and is likely to become progressively automatized. These top-down inputs toward the VWFA possibly influence this region to select relevant bottom-up inputs from lower-level visual areas (represented in pink in the figure) carrying information about the orientation of stimuli. For simplicity inter-hemispheric interactions are not represented here, but it should be acknowledged that during this learning process, local computations in the VWFA can include inhibition of mirror-inversed inputs from the other hemisphere.

Note that although we illustrate it by using mirror-letters (b-d or p-q), our model can eventually be extended to non-mirror letters, such as “e” or “r” for instance, given that each letter has a specific representation at the phonological, gestural (handwriting) and phonatory system. It cannot be excluded however that for these non-mirror letters, the simple extensive visual exposure to their fixed orientation could, in principle, be sufficient to induce visual orientation learning for them. In contrast, this simple passive learning mechanism is unlikely to explain orientation learning for mirror letters given that both mirror representations are regularly present (e.g., b and d). Thus at least for mirror letters, the discrimination mechanism is more likely to involve cross-modal inputs, as represented in our figure. Accordingly, it is known that learning a new set of letters by handwriting produces a better discrimination of its mirror images than when learning by typewriting (Longcamp et al., 2006, 2008). Moreover, despite low performances in pure perceptual visual tasks in mirror discrimination, illiterates are as sensitive as literates in mirror discrimination on vision-for-action tasks (Fernandes and Kolinsky, 2013). Thus, inputs of gestural representations of letters influencing the VWFA perception could have a special weight in the processes of learning mirror discrimination.

It can also be expected that the existence of mirror letters forces the visual system to discriminate them, because it is necessary to correctly read words comprising mirror letters, such as in “bad” (vs. “dad”) for instance. Moreover, evidence suggest that such mirror discrimination sensitivity in literates can be partially generalized to other visual stimuli such as false-fonts (Pegado et al., 2014) and geometric figures (Kolinsky et al., 2011). Thus, it is plausible that during literacy acquisition mirror letters could “drive” the learning process of letter orientation discrimination, eventually extending it for non-mirror letters. Accordingly, in writing systems that do not have mirror letters in their alphabet (e.g., tamil script), even after learning to read and write, literates still present difficulties in mirror discrimination (Danziger and Pederson, 1998). In addition, a superior mirror priming effect for inverted non-mirror letters (e.g., “r”) relative to mirror letters (e.g., “b”) has been reported (Perea et al., 2011), suggesting thus a more intensive automatic discrimination for mirror-letters in comparison to non-mirror letters.

Although it is not known how mirror discriminations of letters and words could be achieved in the complete absence of feedback from phonological, gestural or speech representations, recent empirical and computational modeling work on baboons, who can be trained to acquire orthographic representations in a purely visual manner (Grainger et al., 2012; Hannagan et al., 2014) paves the way to answer this question.

Acknowledging this multi-system interplay during literacy acquisition can have potential implications for educational methods. Interestingly, experiments have suggested that multisensory reinforcement can present an advantage for literacy acquisition: arbitrary print-sound correspondences could be facilitated by adding an haptic component (tactile recognition of letters) during the learning process (Fredembach et al., 2009; Bara and Gentaz, 2011). Large scale studies are now needed to test if promoting multi-system learning is able to provide a clear advantage in real life alphabetization.

### Conflict of interest statement

The authors declare that the research was conducted in the absence of any commercial or financial relationships that could be construed as a potential conflict of interest.
